# The antioxidant protein Oxr1 influences aspects of mitochondrial morphology

**DOI:** 10.1016/j.freeradbiomed.2016.03.029

**Published:** 2016-06

**Authors:** Yixing Wu, Kay E. Davies, Peter L. Oliver

**Affiliations:** MRC Functional Genomics Unit, Department of Physiology, Anatomy and Genetics, University of Oxford, Parks Road, Oxford OX1 3PT, UK

**Keywords:** Oxidative stress, Neurodegeneration, Mitochondria, Mouse, Antioxidant, Neuroprotection

## Abstract

Oxidative stress (OS) and mitochondrial dysfunction are implicated in neurodegenerative disease, suggesting that antioxidant defence systems are critical for cell survival in the central nervous system (CNS). Oxidation resistance 1 (OXR1) can protect against OS in cellular and mouse models of amyotrophic lateral sclerosis (ALS) when over-expressed, whereas deletion of *Oxr1* in mice causes neurodegeneration. OXR1 has emerged therefore as an essential antioxidant protein that controls the susceptibility of neurons to OS. It has been suggested that OXR1 is localised to mitochondria, yet the functional significance of this has not been investigated in the context of neuronal cell death. In order to characterise the role of Oxr1 in mitochondria, we investigated its sub-mitochondrial localisation and demonstrate that specific isoforms are associated with the outer mitochondrial membrane, while the full-length Oxr1 protein is predominately cytoplasmic. Interestingly, cytoplamsic over-expression of these mitochondrially-localised isoforms was still able to protect against OS-induced cell death and prevent rotenone-induced mitochondrial morphological changes. To study the consequences of *Oxr1* deletion *in vivo*, we utilised the *bella* ataxic mouse mutant. We were unable to identify defects in mitochondrial metabolism in primary cerebellar granule cells (GCs) from *bella* mice, however a reduction in mitochondrial length was observed in mutant GCs compared to those from wild-type. Furthermore, screening a panel of proteins that regulate mitochondrial morphology in *bella* GCs revealed de-regulation of phospho-Drp1(Ser616), a key mitochondrial fission regulatory factor. Our data provide new insights into the function of Oxr1, revealing that specific isoforms of this novel antioxidant protein are associated with mitochondria and that the modulation of mitochondrial morphology may be an important feature of its protective function. These results have important implications for the potential use of OXR1 in future antioxidant therapies.

## Highlights

• Oxr1 displays isoform-specific sub-cellular localisation.
• Specific Oxr1 isoforms are associated with the outer mitochondrial membrane.
• Oxr1 can assist in the maintenance of mitochondrial morphology under oxidative stress.

## 1 Introduction

Mitochondrial impairment is a common feature in aging and neurodegenerative diseases [1]. Mitochondria generate reactive oxygen species (ROS) as normal by-products of respiration and the accumulation of ROS can induce oxidative stress (OS) causing damage to critical cellular components such as proteins, lipids and DNA [2]. Furthermore, mitochondrial DNA (mtDNA) damage accumulates with age, triggering the impairment of respiration and ultimately the production of ROS [3]. Mitochondria are also highly dynamic and constantly undergo fusion and fission in order to maintain efficient energy production and mitigate stress; defects in these specific processes can also lead to OS and have been associated with Alzheimer's, Parkinson's and Huntington's disease [4]. However, whether OS is the primary cause or merely a secondary consequence of neurodegeneration still remains unclear [5], and the potential role that OS plays at the presymptomatic stage of neurodegenerative disease requires further investigation. In recent years, factors that are known to counteract OS have been harnessed to alleviate mitochondrial dysfunction in neurodegenerative disease models with mixed success [6]; thus characterising new antioxidant pathways is an important strategy for the identification of novel therapeutic targets.

The protein oxidation resistance 1 (OXR1) was first identified during a screen for genes that were protective against OS [7]. OXR1 has orthologues present in all eukaryotic organisms from yeast to humans and it has been demonstrated that the antioxidant properties of this protein are conserved throughout evolution [7], [8]; *Oxr1* deletion in *Saccharomyces cerevisiae* increases sensitivity to hydrogen peroxide (H_2_O_2_) damage [7] and the mouse *Oxr1* deletion mutant (*bella*) displays progressive ataxia, a shortened lifespan and cerebellar neurodegeneration [9]. More recently, knockdown of OXR1 in mammalian cells was also found to reduce mtDNA integrity and increase the level of cell death under OS [10]. Significantly, increased expression of OXR1 has been shown consistently to be protective both *in vitro* and *in vivo*. For example, in *Caenorhabditis elegans*, over-expression protects cells against OS and aging [11], while over-expression of Oxr1 from *Bombyx mori* significantly extends the life-span of *Drosophila melanogaster* [12]. We have also demonstrated that an Oxr1 transgene can delay neurodegeneration in an mouse model of amyotrophic lateral sclerosis (ALS), [13] and over-expressing Oxr1 in mammalian cells can alleviate cellular abnormalities caused by pathogenic ALS-associated mutations [13]. Taken together, this evidence suggests that OXR1 plays a key protective role against OS.

OXR1 contains a conserved TBC/LysM-associated domain-containing (TLDc) C-terminal domain that is also present in a small family of proteins containing TBC1 domain family, member 24 (TBC1D24) and nuclear receptor coactivator 7 (NCOA7). Significantly, mutations in the TLDc domain of TBC1D24 are associated with Familial Infantile Myoclonic Epilepsy (FIME) and Progressive Myoclonic Epilepsy (PME) [14], [15]. It has also been shown that short isoforms of Oxr1-containing predominantly the TLDc domain - are sufficient to protect cells against OS [9], [16]. The function of the TLDc domain was proposed initially to be enzymatic [17], yet its crystal structure shows a conformation distinct from any other known enzymatic structures [18]. In addition, although there is evidence that Oxr1 is localised to mitochondria in mammalian and yeast cells [9], [19], the function of Oxr1 or the TLDc domain itself remains unknown. Therefore, further understanding of Oxr1 in the context of mitochondrial structure and metabolism may provide valuable clues regarding the function of the protein.

The aim of this study was to determine the functional significance of the sub-cellular and sub-mitochondrial localisation of Oxr1 in the mouse and evaluate the potential effect of *Oxr1* deletion on mitochondrial morphology and metabolism. Our data show that Oxr1 expression in mitochondria is isoform-specific, and that levels of the protein can influence aspects of mitochondrial structure as well as sensitivity to OS.

## 2 Material and methods

### 2.1 Animals

All procedures relating to animal use have been carried out according to UK Home Office regulations and have been approved by the University of Oxford Ethical Review Panel. The *bella* mutation has been described previously as a genomic deletion of approximately 190 kb that spans the entire *Oxr1* locus, thus no protein-coding exons of the full-length isoform (Accession number NM_130885 / Oxr1-FL) or any of the shorter isoforms remain [9]. Homozygous *bella* mutants display progressive ataxia from postnatal day (P)18–20 and disease end-stage, when animals are unable to right themselves, occurs at P22-P24. Animals are maintained on a C3H/HeH background from heterozygous matings and littermate controls were used throughout.

### 2.2 Mitochondria isolation

The purification of mitochondria from tissues or eukaryotic cells was performed as per the manufacturer's instructions (Qiagen).

### 2.3 Mitochondria sub-fractionation

Mitochondria were sub-fractionated by following a swelling-shrinking procedure originally described by Hovius et al. [20] with minor modifications. Isolated mitochondria from tissues were suspended in buffer A (10 μM KH_2_PO_4_, pH 7.4) and placed on ice for 20 min, then 1 volume of buffer B (32% sucrose, 30% glycerol, and 10 mM MgCl_2_, pH 7.4) was added. The mixture was sonicated 6 times for 5 s using a Soniprep 150 sonicator (Jencons-PLS) then centrifuged at 10,000*g* at 4 °C for 10 min. The pellet was re-suspended in buffer A (10 μM KH_2_PO_4_, pH 7.4) and incubated on ice for 20 min. Then, 1 volume of buffer B (32% sucrose, 30% glycerol, and 10 mM MgCl_2_, pH 7.4) was added followed by further sonication as above. The re-suspended pellet was centrifuged at 150,000*g* at 4 °C for 1 h using a Ti70 rotor (Beckman Coulter). Four fractions were obtained corresponding to the outer membrane (OM), the intermembrane space (IMS), the inner membrane (IM) and the matrix (MX). The IMS and MX supernatants were concentrated using Ultracel-10K filters (Millipore). The identity of the fractions was confirmed by western blotting using markers specific mitochondrial compartments: VDAC (1:1000, Cell Signaling) for OM, cytochrome c (1:1000, Cell Signaling) for IMS, cytochrome c oxidase subunit IV (COX IV) (1:2000, Abcam) for IM and HSP-60 (1:1000, Cell Signaling) for MX. The anti-Oxr1 antibody was used at 1:500 [9].

### 2.4 Trypsin susceptibility assay

The trypsin susceptibility assay was carried out as described by Choo et al. [21] with minor modifications. Briefly, increasing concentrations of trypsin (0, 10, 100 and 500 µg/ml) was used to treat isolated mitochondria (150 µg) on ice for 30 min. The mitochondria were then centrifuged at 6000*g* at 4 °C for 10 min for separation from the trypsin solution. Samples were then subjected to western blotting with the Oxr1 antibody and mitochondrial markers as indicted above.

### 2.5 Plasmids

The full-length (NM_130885/Oxr1-FL) or the short mouse Oxr1 isoform (based on NM_001130164 with an additional 28 amino-acid second exon that is shared with Oxr1-FL/Oxr1-C) was cloned into pCMV/myc/cyto (Invitrogen) to generate the myc-tagged cytoplasmic Oxr1 constructs Oxr1-FL-cyto and Oxr1-C-Cyto, respectively. Oxr1-C was targeted to the mitochondria by cloning into pCMV/myc/mito containing an N-terminal mitochondrial targeting signal (MSVLTPLLLRGLTGSARRLPVPRAKIHSL) (Invitrogen) [22] to generate Oxr1-C-Mito. Upon translocation into the mitochondria, the targeting sequence is removed at the N-terminus of Oxr1.

### 2.6 Cell culture and transfection

Cervix epitheloid carcinoma (HeLa) and Neuro2a (N2a) cells were grown routinely in flasks or plates containing DMEM (Life Technologies) supplemented with 10% Foetal bovine serum (FBS) and 1% Penicillin/Streptomycin (Life Technologies) in a humidified incubator at 37 °C in 5% CO_2_. Culturing mouse primary cerebellar granule neurons was conducted as previously described [23]. Transfection was conducted using FuGENE 6 reagent (Promega) as per the manufacturer's instructions.

### 2.7 Cell death assay

HeLa cells expressing Oxr1-myc with cytoplasmic (Oxr1-C-Cyto) and mitochondrial (Oxr1-C-Mito) signals were exposed to 250 μm H_2_O_2_ for 4 h. Cell apoptosis was observed by labelling cells with an anti-cleaved Caspase-3 (Asp175) antibody (1:1400, Cell Signaling). For quantification, five 20× microscopic fields were randomly selected for each coverslip.

### 2.8 Mitochondria quantification

To quantify COX IV-stained mitochondria in cultured primary GCs, confocal images (1024×1024 pixels) were acquired using by a Leica TCS SP5II confocal microscope (63× oil objective; scan speed: 200 Hz) with LAS AF software. Images were then exported and processed with NIH ImageJ FIJI software; the entire range of grey values (0–255) was covered by contrast optimisation, then, binary images of mitochondria were generated. The number of mitochondria per cell and the average area of a mitochondrion were subsequently evaluated using the particle analysing tool (size in pixel units: 10-infinity; circularity: 0.00–1.00).

### 2.9 SDS-PAGE analysis

Protein samples were separated by 12% or 16% SDS polyacrylamide (SDS-PAGE) gel electrophoresis followed by transferring to PVDF membranes (GE Healthcare). Membranes were incubated in blocking solution containing 5% skimmed milk in TBST (10 mM Tris–HCl pH 7.6, 150 mM NaCl and 0.1% Tween 20) for 1 h prior to incubation with primary antibodies for either 1 h at room temperature or overnight at 4 °C. After washing with TBST three times, membranes were incubated with appropriate HRP-conjugated secondary antibodies (1:5000, GE Healthcare). Blots were then developed with the ECL kit (GE Healthcare). Primary antibodies were: rabbit anti-TOM20 (1:200, Santa Cruz), rabbit anti-VDAC (1:1000, Cell Signaling), rabbit anti-cytochrome-c (1:1000, Cell Signaling), rabbit anti-COX IV (1:2000, Abcam), rabbit anti-HSP-60 (1:1000, Cell Signaling), mouse MitoProfile total OXPHOS rodent WB antibody cocktail (1:250, Abcam), mouse anti-c-Myc (1:5000, Sigma-Aldrich), rabbit anti-GAPDH (1:5000, Sigma-Aldrich; or 1:1000, Cambridge Bioscience), mouse anti-Mfn1 (1:250, Abcam), mouse anti-Mfn2 (1:250, Abcam), rabbit anti-Fis1(1:500, Santa Cruz), rabbit anti-Drp1 (1:1000, Cell Signaling), rabbit anti-phospho-Drp1 (Ser616) (1:1000, Cell Signaling), goat anti-OPA1 (1:500, Santa Cruz).

### 2.10 BN-PAGE analysis

Blue Native-PAGE analysis was performed using the NovexH Bis-Tris Gel system (Life Technologies) as per the manufacturer's protocol: isolated mitochondrial samples were lysed in sample buffer containing 2.5% n-dodecyl-b-Dmaltoside (DTT) made using the Native PAGE Sample Prep Kit (Invitrogen). The samples were subjected to centrifugation at 17,000*g* at 4 °C for 30 min and loaded (50 µg) onto a native polyacrylamide Novex 3–12% Bis-Tris Gel (Life Technologies). Electrophoresis (PAGE) was then carried out at 4 °C. After electrophoresis, proteins were transferred to a PVDF membrane (GE Healthcare) and fixed with 8% acetic acid and blocked with 5% skimmed milk in TBST for 1 h. Mitochondrial complexes were probed with monoclonal anti-OxPhos Complex Kit (1:250, Invitrogen).

### 2.11 Immunostaining

Cells (HeLa and primary GCs) on coverslips were washed with PBS followed by incubation in 4% PFA at room temperature for 20–30 min. Coverslips were washed in PBS three times and permeabilised with 0.1% Trition X-100 prior to incubation in blocking solution containing 5% bovine serum albumin (Sigma-Aldrich) and 5% goat serum (Vectorlabs) for 1 h at room temperature. After blocking, coverslips were incubated with primary antibodies for either 1 h at room temperature or overnight at 4 °C. The coverslips were then washed three times in PBS and incubated with secondary antibodies for 2 h (Alexa Fluor, 1:500, Thermofisher). After incubation, coverslips were washed again three times in PBS and mounted in mounting medium with DAPI (Vector Laboratories). Primary antibodies were: mouse anti-c-Myc (1:100, Sigma-Aldrich), rabbit anti-cleaved caspase-3 (Asp175) (1:1400, Cell Signaling), rabbit anti-GABA(A) receptor alpha 6 subunit (1:100, Merck Millipore) and rabbit anti-COX IV (1:1000, Abcam).

### 2.12 Mitochondrial DNA damage test

Mitochondrial damage assay was conducted based on the amplification of long mitochondrial DNA fragments [24]. Total DNA was isolated from cerebella of WT and *bella* mice. Both a long (10.1 kb) and a short mitochondrial fragment (117 bp) were amplified using the following primers: 10.1 kb fragment: 5′-GCC AGC CTG ACC CAT AGC CAT AAT AT-3′ (forward); 5′-GCC AGC CTG ACC CAT AGC CAT AAT AT-3′ (reverse) and 117 bp fragment: 5′-CCC AGC TAC TAC CAT CAT TCA AGT-3′ (forward); 5′-GAT GGT TTG GGA GAT TGG TTG ATG T-3′ (reverse). PCR for the long mitochondrial fragment was carried out using La-Taq DNA polymerase (Takara) with the following PCR conditions: Initial denaturation at 94 °C for 1 min, followed by 24 cycles of 94 °C for 30 s, 60 °C for 30 s and 72 °C for 20 min. PCR for the short mitochondrial fragment was carried out using Taq DNA polymerase (Sigma-Aldrich) with the following PCR conditions: Initial denaturation at 94 °C for 2 min, followed by 35 cycles of 94 °C for 30 s, 56 °C for 30 s and 72 °C for 1 min. The amount of DNA before and after the PCR reactions was quantified fluorometrically using the PicoGreen dsDNA quantitation reagent (Life Technologies). The fluorescent signal was measured at excitation and emission wavelengths of 485 nm and 528 nm, respectively, using a FLUOstar Omega microplate reader (BMG Labtech).

### 2.13 Mitochondrial complex activity assay

Complex I activity assay was conducted based a spectrophotometric method modified from [25]. Firstly, 1 ml of Complex I assay buffer (0.025 M potassium phosphate buffer, pH 7.2, 5 mM MgCl_2_, 13 mM NADH, 65 µM coenzyme Q (Sigma-Aldrich), 0.25% fatty-acid-free albumin, 3.6 μM Antimycin A (Sigma-Aldrich)) was added to a cuvette and warmed to 30 °C. Then, 100 μg of mitochondria were added and mixed with the Complex I assay buffer followed by immediate measurement in a spectrophotometer (Shimadzu) at 340 nm at 30 °C for 3 min. As a control, mitochondrial samples were treated with 10 μM rotenone after the initial measurement and measured again at 340 nm at 30 °C for 3 min. The complex II activity assay was measured by following the protocol as previously described [26], with minor modifications. Firstly, 20 μg purified mitochondria from mouse cerebella were incubated in a cuvette containing assay buffer 1 (0.025 M potassium phosphate buffer, pH 7.2, 5 mM MgCl_2_, 2 mM sodium succinate (Sigma-Aldrich)) at 30 °C for 10 min. Then, 10 μl of buffer 2 (0.025 M potassium phosphate buffer, pH=7.2, 600 µM rotenone, 360 µM antimycin A and 5 mM dichlorophenolindophenol (DCPIP, Sigma-Aldrich)) was added to the cuvette and absorbance at 600 nm was immediately monitored in a spectrophotometer (Shimadzu) for 1 min. Complex III activity was monitored by measuring cytochrome c reduction at 550 nm essentially as described in [27]. Prior to the assay, decylubiquinone was reduced with sodium borohydride (NaBH_4_) to synthesise decylubiquinonol. 20 μg of mitochondria from each sample were incubated in a cuvette of complex III assay buffer (50 mM potassium phosphate, 3 mM sodium azide (NaN_3_), 1.5 μM rotenone, 50 µM cytochrome c (Sigma-Aldrich)). Then, decylubiquinol was added to the cuvette to make a final concentration of 50 µM. Reduction of cytochrome c was read at 550 nm at 30 °C for 1 min. Complex IV activity was carried out by measuring cytochrome c oxidation at 550 nm, a method modified from Wharton et al. [28]. Briefly, approximately 100 μg of mitochondria were added and mixed with the Complex IV assay buffer (50 μM synthesised reduced cytochrome c in 0.1 M potassium phosphate buffer) followed by immediate measurement at 550 nm at 30 °C for 3 min. Citrate synthase activity was assayed by measuring formation of nitrobenzoic acid (TNB) at 412 nm as a result of the reaction between Coenzyme A (CoA-SH) and 5′, 5′-Dithiobis 2-nitrobenzoic acid (DTNB) [29]. In brief, 840 µl of water, 100 µl of assay buffer (1 mM Dinitrobenzoic acid in 1 M Tris–HCl, pH=8.1), 50 µl of synthesised Acetyl CoA and 30 µg of mitochondria were added, mixed and incubated at 30 °C for 5 min. After incubation, 50 µl of 10 mM Oxaloacetate (Sigma-Aldrich) was added followed by immediate measurement at 412 nm at 30 °C for 1 min.

### 2.14 Mitochondrial stress test

Mitochondrial stress testing was carried out using a Seahorse Bioscience XF96 analyser (Seahorse Bioscience Inc.) in 96-well plates at 37 °C as per the manufacturer's instructions with minor modifications. Briefly, GCs at P7 were seeded at 2×10^5^ cells/well one week prior to the assay. On the test day, the growth media was removed, washed once and replaced with XF assay media (Seahorse Bioscience Inc.) and the plate was incubated in a CO_2_-free incubator for 1 h at 37 °C. The hydrated cartridge sensor was loaded with appropriate volume of mitochondrial inhibitors to achieve final concentrations in each well: oligomycin (3 µM), carbonilcyanide p-triflouromethoxyphenylhydrazone (FCCP) (2 μM) and with rotenone/antimycin A (both 2 µM). Then, levels of basal respiration, ATP production, proton leak, maximal respiration and non-mitochondrial respiration were measured as described in manufacturer's protocol.

### 2.15 Transmission Electron Microscopy (TEM)

Perfused brain tissue was prepared according to standard TEM protocols [30]. Blocks were sectioned using a UC7 ultramicrotome (Leica) and a Diatome diamond knife. Sections (90 nm) were post-stained with lead citrate and imaged on a Tecnai 12 TEM (FEI) operated at 120 kV. Digital images were acquired using a US1000 CCD camera (Gatan) and OneView (Gatan).

### 2.16 Statistical analysis

Results were analysed using GraphPad Prism software. Pairwise analyses between wild-type and mutant or between the various treatments were compared using a 1-way ANOVA with post-hoc analysis or Student's *t*-test as indicated. *P*-values <0.05 were considered to be significant. Data are expressed as the mean −/+ S.E.M.

## 3 Results

### 3.1 Different isoforms of Oxr1 are expressed in specific subcellular compartments

**Fig. 1:**
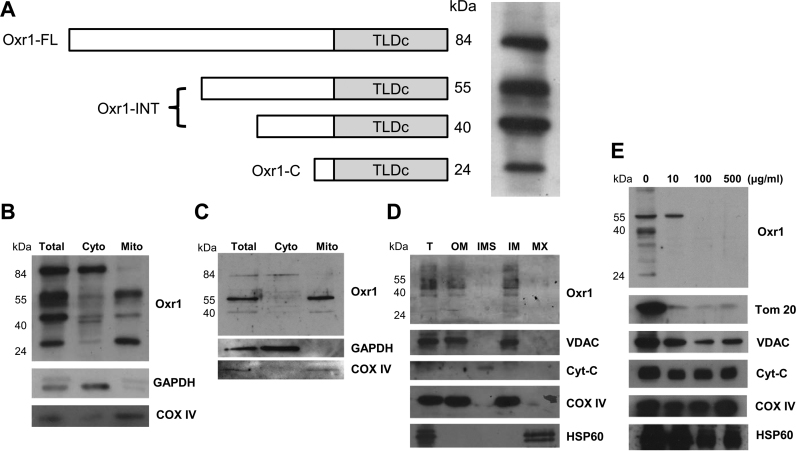
Oxr1 isoforms display differential cellular localisation patterns. (A) A schematic illustration of four predicted Oxr1 isoforms in mouse detected by western blot in this study: Oxr1 full-length (FL) at approximately 84 kDa, intermediate isoforms (INT) at approximately 55 kDa and 40 kDa, and the shortest (C) isoform at approximately 24 kDa. Total brain homogenate from wild-type mouse brain was blotted with anti-Oxr1 [9]. (B, C) Total tissue homogenate, cytoplasmic and mitochondrial fractions from wild-type mouse brain (B) and spinal cord (C) were subjected western blot analysis with anti-Oxr1, anti-GAPDH as a cytoplasmic marker and anti-COX IV as a mitochondrial marker. (D) Mitochondria purified from wild-type mouse cerebellar tissue were sub-fractionated into OM, IMS, IM and MX by ultracentrifugation. Each sub-fraction in addition to the total mitochondrial sample (*T*) were subjected to western blotting using the anti-Oxr1 antibody and specific markers for individual compartment as described in the Material and Methods. Oxr1 is mainly detected in OM and IM fraction. (E) Mitochondria purified from wild-type mouse brain were incubated with increasing concentrations of trypsin prior to immunoblotting with mitochondrial marker proteins: TOM20 and VDAC for the OM, cytochrome c (Cyt-C) and COX IV for the IMS and IM, respectively and HSP-60 for MX. All wild-type tissue used was from male C3H/HeH mice at P56.

Several isoforms of Oxr1 have been described to date in mammalian cells, and evidence from cDNA cloning, qRT-PCR and western blotting suggest that all of these contain the conserved TLDc domain (Fig. 1A) [9], [16]. However, the subcellular localisation of these individual isoforms has not been investigated. To address this important question, total tissue homogenates, cytosolic and mitochondrial fractions from wild-type mouse brain and spinal cord were subjected to western blotting using an antibody against an epitope in the TLDc domain of Oxr1 [9]. In total tissue lysate, four main isoforms of Oxr1 are detected in mouse brain at approximately 84, 55, 40 and 24 kDa (Fig. 1A). Upon fractionation, however, the shortest isoform (Oxr1-C, approximately 24 kDa) was almost exclusively localised in the mitochondrial fraction from the brain (Fig. 1B), while the full-length Oxr1 isoform (Oxr-FL, approximately 84 kDa) was localised in the cytosolic fraction. In addition, the two intermediate isoforms were enriched in the mitochondrial fraction. In the spinal cord, a similar distribution was observed, although Oxr1-C could not be detected (Fig. 1C). These data suggest that some, but not all, Oxr1 isoforms are localised to the mitochondria.

### 3.2 Mitochondrially localised Oxr1 is associated with mitochondrial OM

We next studied the sub-mitochondrial localisation of Oxr1 using ultracentrifugation to separate the outer membrane (OM), the intermembrane space (IMS), the inner membrane (IM) and the matrix (MX) prior to immunoblotting. Interestingly, these data indicate that the shorter isoforms of Oxr1 are localised in the mitochondrial OM and IM, while only a very small proportion of these isoforms was detected in the IMS and MX (Fig. 1D). In addition, the specificity of the sub-fractionation was confirmed using a set of specific markers (Fig. 1D). It is noted that the OM marker voltage-dependent anion channel (VDAC) was also observed in the IM, while the IM marker COX IV was also detected in the OM fraction; this is likely due to incomplete OM/IM separation due to multiple contact points between mitochondrial membranes as previously described [21], [31].

To further characterise the membrane-associated localisation of Oxr1 within mitochondria, a trypsin susceptibility assay was conducted using intact mitochondria purified from mouse brain [21] (Fig. 1E). All Oxr1 isoforms were digested at the lowest (10 μg/ml) trypsin concentration, apart from the 55 kDa isoform that required 100 µg/ml (Fig. 1E). As a positive control, TOM20-a receptor loosely associated with the cytosolic side of the OM [32]-was assayed in parallel, and this appeared to have poor resistance to trypsinisation as expected. Conversely, VDAC and COX IV- transmembrane proteins localised in OM and IM, respectively – were not digested even in the presence of high concentration (500 µg/ml) of typsin (Fig. 1E). Furthermore, cytochrome c and HSP60-localised in IMS and MX, respectively – were also barely affected (Fig. 1E). Taken together, these data suggest that the shorter isoforms of Oxr1 are loosely associated with the outer mitochondrial membrane, potentially facing towards the cytosol.

### 3.3 Over-expression of Oxr1 in the cytosol is more protective against oxidative stress-induced cell death than mitochondrial over-expression

**Fig. 2:**
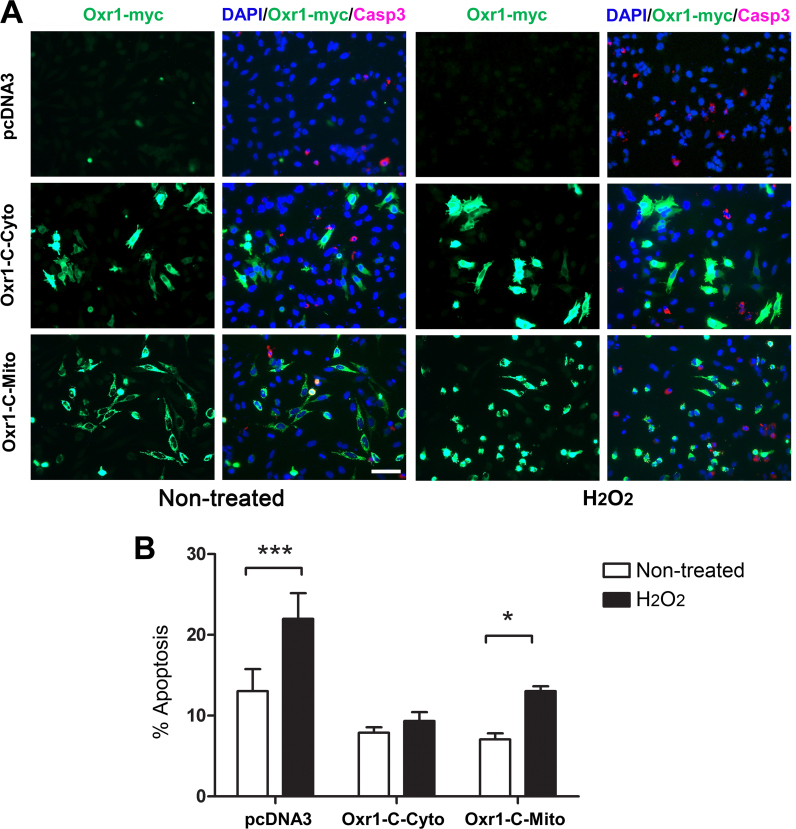
Cytoplasmic over-expression of Oxr1 confers stronger protection against oxidative stress than mitochondrial over-expression. (A) Representative images of HeLa cells transfected with the control vector (pcDNA3), Oxr1-C-Cyto or Oxr1-C-Mito in non-treated (NT) conditions or after H_2_O_2_ treatment (50 μM for 4 h). Cells were immunostained with anti-myc (for Oxr1) or anti-cleaved caspase-3 (Casp3) to quantify apoptosis. Scale bar=50 µm. (B) Cells expressing Oxr1-C-Cyto show stronger protection against oxidative insult in comparison with cells expressing Oxr1-C-Mito or the pcDNA3 control vector. Data are shown ±SEM of 4 independent experimental repeats and analysed by ANOVA followed by Bonferroni post-hoc tests, *p<0.05, ***p<0.001.

We have shown previously that the Oxr1-C isoform, which contains almost exclusively the TLDc domain, is capable of preventing oxidative damage *in vitro* [9]. Indeed, this isoform appears to be as potent an antioxidant as the full-length isoform (Oxr1-FL) in neuronal cells [33], suggesting that the TLDc domain alone is able to confer neuroprotection. It is noteworthy, however, that these experiments have used a promoter that expressed the cloned Oxr1-C protein in the cytoplasm [9]. Having demonstrated here that non-full-length isoforms of Oxr1 are likely to be loosely associated to the mitochondrial membrane, we therefore wished to investigate whether cytoplasmic over-expression of this particular Oxr1-C isoform was more neuroprotective against OS compared to targeted over-expression in mitochondria, where is it found endogenously. Cells were either transfected with Oxr1-C containing a standard CMV promoter with an additional N-terminal mitochondrial localisation signal (MLS) (Oxr1-C-Mito), Oxr1-C without an additional MLS tag (Oxr1-C-Cyto) or an empty negative control vector. First, the sub-cellular localisation of each Oxr1-C protein was confirmed by co-immunostaining with appropriate markers (Supplementary Figure 1A). In addition, the expected localisation of the tagged proteins was confirmed by fractionation of transfected cells and western blotting. (Supplementary Figure 1B). To induce OS, cells were treated with hydrogen peroxide (H_2_O_2_) and cell death was measured by quantifying cleaved caspase-3, a marker of cell apoptosis, compared to untreated cells (Fig. 2A). After treatment, cells transfected with the control vector showed an increase in the proportion of cell death of approximately 80% (Fig. 2B). In line with previous results, expression of the cytoplasmic Oxr1-C-Cyto construct was protective against cell death, and after treatment there was only an approximately 20% increase in cell death, significantly lower than the control vector (Fig. 2B). Interestingly, mitochondrial over-expression of Oxr1-C (Oxr1-C-Mito) did not appear to show any protection against OS as the percentage increase in cell death was similar to the control vector (an approximately 90% increase) (Fig. 2B). This indicates that cytoplasmic over-expression of Oxr1-C is potentially more effective at preventing OS-related cell death than mitochondrial over-expression.

### 3.4 Cytoplasmic but not mitochondrial over-expression of Oxr1 alleviates mitochondrial morphological changes caused by acute rotenone-induced oxidative stress

**Fig. 3:**
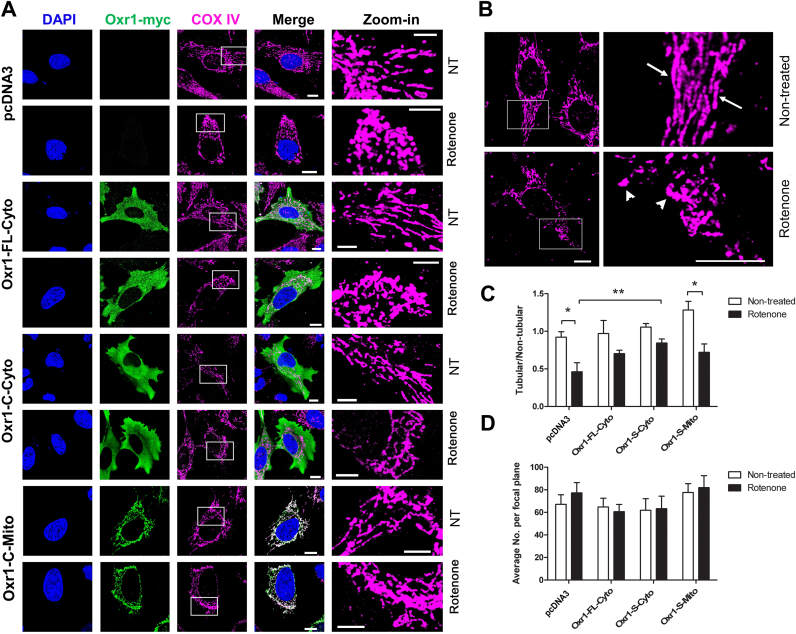
Cytoplasmic over-expression of Oxr1 alleviates mitochondrial morphological changes induced by acute rotenone treatment. (A) Representative images showing HeLa cells transfected with the control vector, Oxr1-FL-Cyto, Oxr1-C-Cyto or Oxr1-C-Mito, in non-treated (NT) conditions or under rotenone treatment (50 μM, 1 h) conditions; Cells were immunostained with anti-myc (for Oxr1) and anti-COX IV antibodies. Scale bar=20 µm. (B) Representative images showing tubular mitochondria in untreated control cells (arrows), while non-tubular (donut/blob) mitochondria become predominant after rotenone treatment (arrowheads). Scale bar=10 µm. (C) Ratio of mitochondria classified as tubular versus non-tubular before and after rotenone treatment. Rotenone treatment significantly reduced the ratio of tubular to non-tubular mitochondria, but over-expression of cytoplasmic Oxr1 prevented this reduction. (D) The total number of mitochondria per focal plane remains unchanged after rotenone treatment. Data are shown as the mean±SEM from 3 independent replicates of each genotype. A minimum of 15 cells were analysed per group in each repeat. Data are shown as mean±SEM and were analysed by ANOVA followed by Bonferroni post-hoc tests; *p<0.05, **p<0.01.

It is well-established that mitochondrial morphology is modified in response to oxidative stress and that these changes can subsequently affect mitochondrial function [34], [35], [36]. As Oxr1-C was able to provide protection against OS, we wanted to further understand the potential role of mitochondria in this process. Therefore, we examined whether over-expressing Oxr1 prevents mitochondrial morphological changes induced by OS. For these experiments, we selected rotenone (50 µM for 1 h) as a specific and potent inhibitor of mitochondrial complex I capable of causing oxidative damage and mitochondrial morphological changes [34], [37], [38]. Prior to treatment, cells were transfected with either an empty vector, Oxr1-C-Cyto or Oxr1-C-Mito. We also added an Oxr1-FL-Cyto construct to this experiment to complement our other studies that have utilised successfully this full-length isoform as a protective protein *in vivo* [9], [39] (Fig. 3A). Consistent with previous findings [34], by quantifying mitochondria labelled with COX IV, rotenone exposure caused a shift of mitochondrial shape from mainly tubular, which is found in healthy non-treated cells, to non-tubular (‘donut’ or ‘blob’ shape) (Fig. 3B); thus in cells transfected with empty vector, the ratio between tubular and non-tubular mitochondria was significantly reduced by over 2-fold after rotenone exposure (Fig. 3C). In cells over-expressing Oxr1-FL-Cyto or Oxr1-C-Cyto, no significant reduction of this ratio was observed, suggesting a protective role of cytoplasmic Oxr1 against mitochondrial morphological modification induced by rotenone (Fig. 3C). Interestingly, however, expression of the Oxr1-C-Mito construct caused a shift towards non-tubular mitochondria, similar to that observed for cells transfected with an empty vector. In addition, pair-wise comparisons showed that under rotenone treatment, only cells over-expressing Oxr1-C-Cyto had a significantly higher proportion of tubular mitochondria compared to control cells (Fig. 3C). Next, to exclude the possibility that these ratios were confounded by a change in total number of mitochondria after treatment, the total number of mitochondria per focal plane was quantified, and no differences were observed between the experimental groups (Fig. 3D). In summary, these results suggest that cytosolic over-expression of either Oxr1-FL or Oxr1-C is more effective at alleviating mitochondrial morphological changes caused by rotenone than over-expression of Oxr1-C in mitochondria.

To further investigate these findings in relation to endogenous Oxr1, we examined whether there would be a shift in the ratio between the mitochondrial and cytoplasmic distribution of Oxr1 during OS. N2a cells were subjected to a timecourse of H_2_O_2_ treatment, followed by fractionation and western blotting. N2a cells express all the Oxr1 isoforms found in mouse brain and these data show the expected presence of the shortest isoform (~24 kDa, Oxr1-C) in the mitochondria and the longest (Oxr1-FL) in the cytoplasm (Supplementary Figure 2A). Specifically, quantification of the shortest isoform revealed there was a trend towards a reduction in mitochondrial expression levels after OS, yet this was not significant, and there was no detectable appearance of this specific isoform in the cytoplasmic fraction, even up to 2 h after H_2_O_2_ treatment (Supplementary Figure 2B). In addition, the expression of other Oxr1 isoforms did not significantly alter under OS. These data suggest that there is no overt shift in the sub-cellular distribution of Oxr1 from the mitochondria to the cytoplasm under these OS conditions.

### 3.5 Mitochondrial length is reduced in primary cerebellar GCs from mice lacking *Oxr1*

**Fig. 4:**
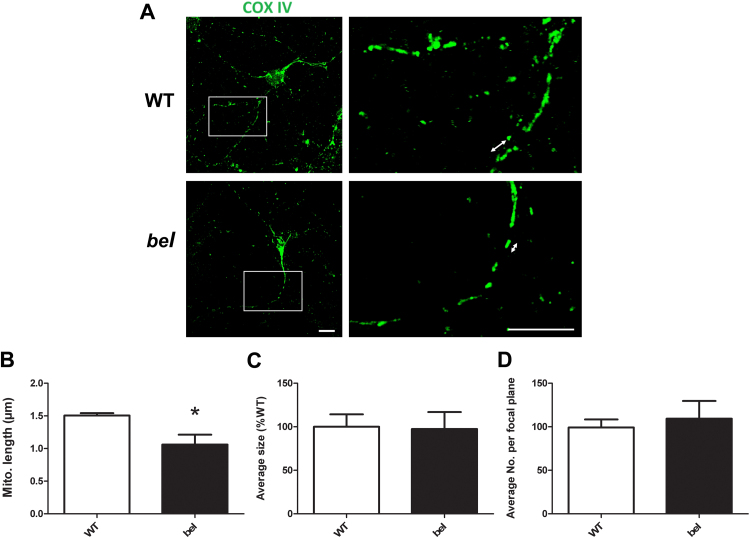
Mitochondrial morphological changes in primary cerebellar GCs of *bella* mice. (A) Representative images showing mitochondria in wild-type (WT) and *bella* (*bel*) primary cerebellar GCs. Scale bars = 10 µm. (B) Mitochondrial length (example arrows in (A)) is significantly reduced in *bella* compared to WT GCs. (C and D) No difference was observed in the average size or total number of mitochondria per focal plane between WT and *bella* GCs. Mitochondria were quantified in primary GCs of 3 WT and 3 *bella* mice and a minimum of 10 cells were analysed per replicate. Data are shown as mean±SEM and analysed by Student's *t*-test; *p<0.05.

Our data suggest that over-expressing cytosolic Oxr1 *in vitro* is able to alleviate OS-induced mitochondrial morphological changes; thus we speculated that the complete loss of *Oxr1* would influence mitochondrial structural integrity or function. To examine this hypothesis, we ultilised primary cerebellar GC neurons from the homozygous *bella* mutant mouse (*Oxr1*^*bel/bel*^), that does not express *Oxr1* due to deletion of the entire genomic locus [9]. Cerebellar GCs are the only region of the brain where neurodegeneration is detected at disease end-stage in *bella* mutants, suggesting that these particular cells are more susceptible to deletion of *Oxr1* than other neuronal populations. Primary GCs for these experiments from *bella* mice and wild-type (WT) littermate controls at P7 were isolated and grown *in vitro* for 7 days (DIV7) and their identity confirmed with GC marker GABA(A) receptor subunit alpha-6 (GABRA6) (Supplementary Figure 3). By quantification of mitochondrial structure between the two genotypes, we discovered that mitochondrial length was significantly reduced in primary GCs of *bella* mice in comparison with WT (Fig. 4A and B). Importantly, there was neither a difference in average size or total number of mitochondria per focal plane between the genotypes (Fig. 4C and D); these data suggest that the mitochondrial population shifts towards a more non-tubular structure when *Oxr1* is deleted. Thus to begin to test whether loss of *Oxr1* can lead to modifications in mitochondrial ultrastructure *in vivo*, transmission electron microscopy (TEM) using ultra-thin sections was conducted to study cerebellar tissue from near end-stage (P20) *bella* and age-matched WT mice. Mitochondria from multiple regions in the GC layer were assessed, and both genotypes displayed normal and comparable mitochondrial morphology (Supplementary Figure 4). Taken together, these results suggested that deletion of *Oxr1* in mice can alter the gross morphology of mitochondria towards a shape indicative of OS, but does not cause any overt abnormalities in ultrastructure *in vivo*.

### 3.6 Deletion of *Oxr1* influences expression of mitochondrial fission regulators

**Fig. 5:**
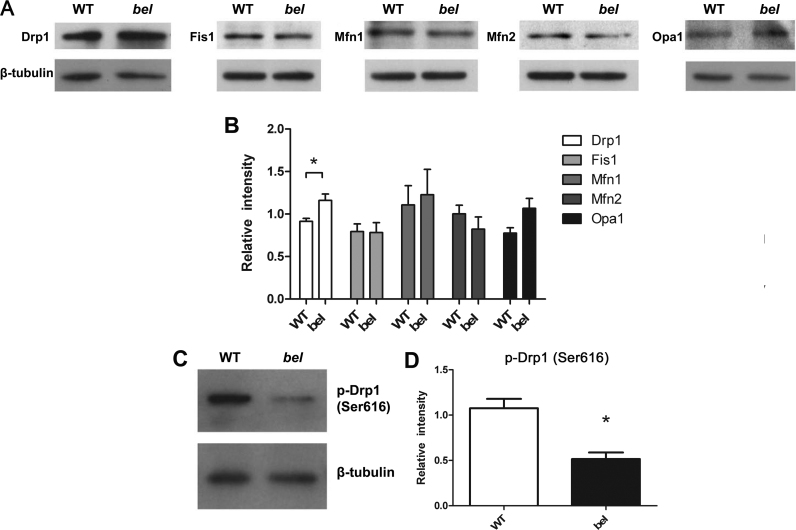
Expression level of mitochondrial fusion and fission proteins in the cerebellum of WT and *bella* mice. (A) Cerebellar homogenates from end-stage (P22) *bella (bel)* and age-matched wild-type (WT) mice were subjected to western blotting analysis for mediators of mitochondrial fission (Drp1 and Fis1) and fusion (Mfn1, Mfn2 and Opa1). (B) Quantitative analysis by densitometry; the total level of Drp1 is significantly higher in *bella* compared to WT tissue. (C) Western blot of cerebellar homogenate from end-stage *bella* and age-matched WT mice probed for p-Drp S616, indicating that the expression level in *bella* is significantly lower than WT (D). Data are shown as mean±SEM from 3–6 independent samples of each genotype and were analysed by Student's *t*-test; *p<0.05.

Since mitochondrial shape and morphology are regulated by fusion and fission [40], we examined next whether deletion of *Oxr1* affects these processes. Protein levels of major known fusion regulators, Mfn1, Mfn2 and OPA1 as well as fission mediators Drp1 and Fis1 were quantified from WT and *bella* cerebellar homogenates taken at P22 (Fig. 5A). No differences in expression were detected between genotypes, with the exception of Drp1, that showed a small but statistically significant increase in *bella* mice compared to WT (Fig. 5B). Under OS, Drp1 is phosphorylated at Ser616, which activates the protein for recruitment to mitochondria to facilitate the fission process; conversely, dephosphorylation at Ser616 inhibits mitochondrial fission [41], [42]. We hypothesised that there may be an inhibitory effect on the activity of Drp1 in mutant mice, thus we tested the level of phospho-(p-)Drp1 S616 (Fig. 5C). Interestingly, the level of p-Drp1 S616 was significantly lower in cerebella homogenate from *bella* compared to WT mice (Fig. 5D), indicating an inhibitory effect of Drp1's activity. Overall, these data suggest that the change in mitochondrial morphology observed in *bella* mice may be caused by a compensatory pathway that inhibits the activity of Drp1 through dephosphorylation of Ser616 in response to stress caused by the deletion of *Oxr1*.

### 3.7 Deletion of *Oxr1* does not affect mitochondrial DNA integrity or ETC protein activity

**Fig. 6:**
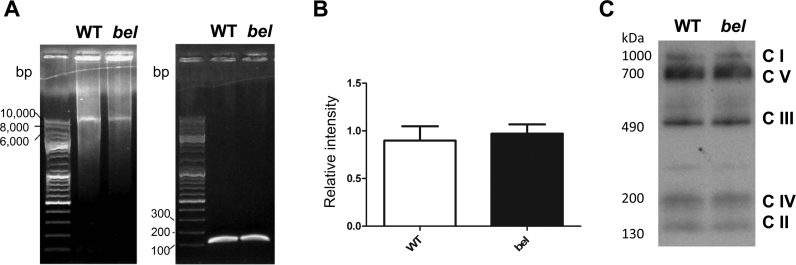
No evidence for mtDNA damage or mitochondrial complex assembly defects in *bella* mice. (A) Representative gel images showing PCR products of long (10.1 kb) and short (117 bp) mtDNA fragments from total DNA extracted from cerebellar tissue of end-stage *bella (bel)* and age-matched WT mice. (B) Quantification of mtDNA (long fragment) was carried out using the PicoGreen method by normalisation to the short mtDNA fragment. Data are shown as the mean ± SEM from 5–6 replicate samples of each genotype and analysed by Student's *t*-test. (C) End-stage *bella* and age-matched WT cerebellar tissue were subjected to BN-PAGE analysis to resolve purified mitochondrial proteins solubilized by the detergent n-dodecyl-β-d-maltopyranoside (DDM). A representative image is shown of samples probed using an anti-OxPhos Complex antibody indicating no defects in complex assembly in *bella* mice.

The vulnerability of mitochondrial (mt)DNA to OS has been well-established [43], [44] and recently, accumulation of mtDNA damage was found in OXR1 depleted (approximately 85% knockdown) HeLa cells treated with H_2_O_2_ [10]. Furthermore, we have shown previously that OS-induced DNA damage occurs in the cerebellar GC layer of end-stage *bella* mice [9]. To study next whether the deletion of *Oxr1* can cause accumulation of endogenous mtDNA lesions in *in vivo*, DNA was extracted from the cerebellum of WT and end-stage (P22) *bella* mice and the mtDNA was subjected to quantitative (q)PCR (Fig. 6A). Using this method, no increase in mtDNA lesions was evident in the *bella* cerebellum compared to WT (Fig. 6B).

Previous discoveries have suggested an association between mitochondrial oxidative phosphorylation (OXPHOS) abnormalities and neurodegenerative disease [45]. Therefore, to examine whether the neurodegeneration observed in *bella* mice is related to such defects, we first examined ETC complex protein expression and assembly. Initially, the expression level of five ETC complex subunits (Complex (C) I to V) in both WT and *bella* mice were measured simultaneously by western blot and no difference in between genotypes was observed (Supplementary Figure 5). We then tested whether loss of *Oxr1* had an impact on mitochondrial complex assembly pathways by conducting Blue Native (BN) PAGE analysis to detect formation of the five ETC complexes from cerebellar tissue. These data also revealed no difference between WT and *bella* mice (Fig. 6C). In addition, the mitochondrial ETC complex activities of intact mitochondrial extracts from the cerebella of end-stage *bella* and age-matched WT mice were measured. However, no difference in CI, CII, CIII, CIV or citric synthase activity was observed between genotypes (Supplementary Figure 6).

### 3.8 Primary cerebellar GCs from *bella* mice display normal mitochondrial metabolism

**Fig. 7:**
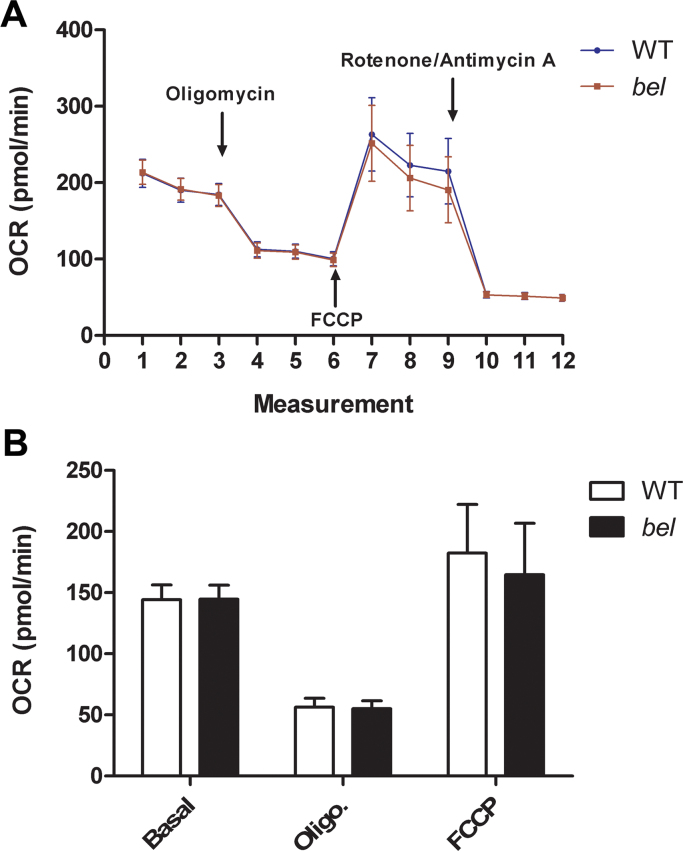
Oxygen consumption in primary GCs is not affected by deletion of *Oxr1*. (A) Oxygen consumption rate (OCR) profiles are expressed as pmol of oxygen consumed per minute in primary GCs of wild-type (WT) and *bella (bel)* mice. Arrows indicate when mitochondrial inhibitors/stressors were added to the assay: oligomycin (3 µM), FCCP (2 µM) and rotenone and antimycin (2 µM, each). (B) Normalised OCRs after subtraction of rotenone/antimycin treated OCR (non-mitochondrial respiration), under basal condition, after adding oligomycin (3 µM, proton leak) or FCCP (2 µM, maximal respiration). The data were obtained from 3 independent experiments using primary GCs obtained from 6 WT and 5 *bella* mice. Data are shown as the mean±SEM.

To date, loss or disruption of a number of antioxidant proteins has been found to influence mitochondrial oxygen consumption as part of a potential neurodegenerative pathway [46], [47]. We therefore studied whether deletion of *Oxr1* can cause similar defects by measuring mitochondrial oxygen consumption and other respiratory parameters from WT and *bella* primary GCs cultured for 7 days. Basal respiration level was first measured prior to addition of any mitochondrial stressors, and no difference was observed between genotypes (Fig. 7A). After adding the mitochondrial complex V inhibitor oligomycin, a marked decline of oxygen consumption level (approximately 100 pmol/min) was observed in both WT and *bella* GCs*,* indicating the same amount of oxygen was used for ATP production. Next the mitochondrial uncoupler FCCP was added, triggering a marked induction of oxygen consumption (approximately 150 pmol/min) in both WT and *bella*, suggesting the spare oxygen consumption capacity of GCs from both genotypes was equivalent (Fig. 7A). Finally, rotenone and antimycin A, which inhibit CI and CIII respectively, were added to the assays resulting in a considerable reduction in oxygen consumption as expected. The remaining oxygen consumption level indicated the amount of oxygen available for non-mitochondrial respiration, at approximately 50 pmol/min for both genotypes. By subtracting the non-mitochondrial oxygen consumption level, the normalised data suggest that there was no significant difference of mitochondrial oxygen consumption in basal level, proton leak stage or maximum respiration between WT and *bella* GCs (Fig. 7B).

## 4 Discussion

In this study we have demonstrated that Oxr1 is expressed in an isoform-specific pattern in mitochondria and that the expression levels of this novel antioxidant protein are likely to play a role in regulating aspects of mitochondrial morphology in the presence of OS.

Several isoforms of OXR1 and its orthologues have been described in mammalian and non-mammalian systems [7], [19], [48], and western blot results have indicated a complex differential distribution of isoforms in mouse tissue [9], [13]. Here, we reveal the cytosolic localisation of the longest Oxr1-FL (84 kDa) isoform and mitochondrial enrichment for the shorter isoforms (55, 40 and 24 kDa) based on our antibody raised against a shared C-terminal epitope. These findings suggest that the mitochondrial localisation and induction of OXR1 observed previously in mammalian cells may have originated from non-full-length isoforms [9], [19]. In addition, using a combination of ultracentrifugation and trypsin digestion assays, our data suggest that that Oxr1 is not likely to be present in the mitochondrial matrix but is found at the mitochondrial OM, facing the cytosol. It is still unclear, however, how Oxr1 is translocated to this position. It has been postulated that a mitochondrial localisation signal (MLS) is present at the N-terminus of Oxr1 in yeast (scOxr1) [19], yet no such region is present in any of the known isoforms of OXR1 in mouse or human. Indeed, in our study, while tagged mouse Oxr1 proteins with an additional MLS are successfully targeted to mitochondria (Oxr1-C-Mito), over-expression of constructs with no additional MLS (both Oxr1-C-Cyto and Oxr1-FL-Cyto) are not mitochondrially localised. Interestingly, we were also unable to target the full-length Oxr1 protein into mitochondria using the same N-terminal MLS (data not shown). These findings are thus consistent with a transient association between Oxr1 and mitochondria that may be size-selective. Of note, the anti-oxidative protein DJ-1, mutated in familial Parkinson's disease, has a similar OM-associated localisation, despite lacking a *bona fide* MLS [49], [50], [51], [52]. Translocation of DJ-1 into mitochondria is thought to be mediated by interacting with chaperones including heat-shock protein (HSP) 70, C-terminus of heat-shock cognate 71 kDa protein (HSC70)-Interacting Protein (CHIP) and mtHsp70/Grp75 [24]. It is possible that the translocation of Oxr1 to mitochondria occurs via a similar mechanism; for example, our initial results from a protein interaction screen suggest that under OS, Oxr1-C may interact with DNAJC19 [13], a mitochondrial protein translocase component [53]. Further studies will be required to confirm such associations and understand their functional relevance to OS sensitivity.

Our cell survival assays revealed that cytoplasmic expression of the shortest Oxr1 isoform (Oxr1-C-Cyto), containing almost exclusively the TLDc domain, is protective against OS-induced apoptosis; this is consistent with previous findings that the TLDc domain of Oxr1 is a key feature of its anti-oxidative role [9], [33]. Interestingly, more effective protection against OS-induced apoptosis was conferred by over-expressing Oxr1-C in the cytoplasm compared to targeting the same isoform to the mitochondria. Importantly, the MLS used here was from subunit VIII of human cytochrome c oxidase that targets proteins to the mitochondrial matrix [22]; thus our findings suggesting that a subtle balance between cytosolic and mitochondrial OM localisation may be required for the anti-oxidative role of Oxr1 in these assays. Interactions of various pro- and anti-apoptotic proteins as well as ROS sensors occur at the interface between the cytosol and the mitochondrial OM, and these binding events are thought to mediate mitochondrial OM permeabilisation (MOMP) one of the hallmarks of the early stage of apoptosis [54]. Moreover, regulation of MOMP is independent from IM and MX proteins [55]. Hence, it will be worthwhile to determine whether the OM-associated, cytosolic-facing Oxr1 isoforms establish their anti-oxidative role via these pathways.

Interestingly, we did not see an induction or significant shift in the subcellular re-localisation of Oxr1 isoforms in N2a cells under OS. Previous data have indicated that certain isoforms of OXR1 can be induced under stress at the protein level [19]; yet other studies indicate that this response may be transient and cell-type specific, as shown by temporal, isoform-specific induction [13], [33] or a lack of induction [33], [56] at the RNA level under OS. The fact that neither the shortest or longest *OXR1* isoforms lack a predicted or experimentally determined antioxidant response element (ARE), supports the notion that the gene is not downstream of classical Nrf2-mediated OS-response pathways [57], [58]; however, further work is needed to determine the complexities of the timing and functional significance of endogenous Oxr1 induction to cellular survival.

In an earlier study, Elliot et al. over-expressed human OXR1 with an additional MLS in a mutant strain of yeast that is sensitive to H_2_O_2_-induced lethality and their data suggested that mitochondrial localisation of OXR1 is required for protection against OS [19]. The apparent discrepancy between these results and our new study may relate to differences in the type of assay or species used, the levels of over-expression achieved, or the type of MLS used to tag OXR1 itself; although Elliot et al. used a MLS from yeast Sod2 that should also direct proteins to the mitochondrial matrix. The particular isoforms of Oxr1 used in such OS protection assays must also be considered. We have focused here on the shortest Oxr1-C isoform that is based on a known cDNA that is expressed in mouse and humans [9] that we have also shown to be neuroprotective [9], [33]. Whereas other studies have used longer isoforms containing additional exons outside of the TLDc domain – likely to be represented by the 44 and/or 55 kDa proteins we see by western blot – that may also play an important role in OS resistance [16].

Oxr1-related proteins localised to other cellular compartments may also confer protection against OS-related insults; for instance over-expression of Oxr1 from the silk-worm *Bombyx mori* (BmOxr1) was protective against OS in *Drosophila melanogaster* [12]. Interestingly, when over-expressed, BmOxr1 was found mainly in the nuclei of *Drosophila melanogaster* cells. The sequence alignments provided suggest that this BmOXR1 protein has sequence identity with NCOA7, a known nuclear-expressed TLDc domain-containing protein that is also able to confer protection against OS [8].

Mitochondrial morphological changes caused by rotenone have been demonstrated to be OS-related [34]. We show here that over-expression of Oxr1 in the cytoplasm is capable of preventing the mitochondrial morphological changes induced by rotenone, while a reduction of mitochondrial length is observed in primary GCs lacking *Oxr1*. These data are in line with our recent observations that Oxr1 can prevent mitochondrial morphology defects caused by ALS-associated Tdp-43 mutations [13]. Taken together, these studies further suggest that Oxr1 is involved in maintaining mitochondrial morphology in the stress response as part of its antioxidant function. Mitochondrial morphology is intensively regulated by fusion and fission processes [40] and multiple lines of evidence have shown that under OS, once phosphorylated at Ser616, the fission protein Drp1 is activated to facilitate mitochondrial fragmentation [41], [59]. Based on our data from primary cells, we hypothesised that we might observe a similar shift in the mitochondrial population towards a more pro-fission status in cerebellar tissue of end-stage *bella* mice. Unexpectedly, however, the level of p-Drp1 (Ser616) was found to be reduced significantly in *bella* cerebellar tissue compared to WT. This could be a result of a compensatory effect of Drp1 phosphorylation due to cellular stress; indeed, a reduction of p-Drp1 S616 is also observed in cells upon starvation [60].

As we observed mitochondrial structural changes in cells lacking *Oxr1*, we also examined whether any other hallmarks of mitochondrial dysfunction could be detected. No defects in oxygen consumption or other respiratory measures were identified in primary GCs from *bella* mice, and there was no evidence for mtDNA damage or ultrastructural abnormalities in cerebellar tissue from the same mutants. Since our previous results show that only a small percentage of neurons in the cerebellar granule cell layer are affected in end-stage *bella* mutants [9], our data presented here may reflect that overall mitochondrial functional differences in a large population of cells or tissue lacking *Oxr1* are simply below the detection threshold of even sensitive quantitative assays. Moreover, several lines of evidence indicate that the function of OXR1 becomes more critical, or key protective pathways become activated, under OS conditions. For example, an increase in mtDNA damage was observed previously in *OXR1* knockdown cells, although exogenous OS was required to exacerbate this effect [10]. In addition, an recent RNA-seq study in the same HeLa cell model revealed additional cell death-response pathways were activated when OXR1 knockdown and OS were combined [56].

## 5 Conclusions

In summary, our study reveals the detailed sub-cellular and sub-mitochondrial localisation patterns of Oxr1 isoforms and proposes that this protein plays a role in modulating mitochondrial morphology. In recent years, delivering antioxidant proteins has been considered as a potentially effective way of treating disease where OS is thought to be an important contributing factor to pathology [61], [62]. Indeed, targeting OXR1 to mouse kidney has been shown to be protective against experimentally-induced renal injury and inflammation [61] and mutations in an *Oxr1* homologue in *Drosophila melanogaster* show increased tolerance to *Vibrio cholerae* infection [63]. Our findings deepen the understanding of Oxr1 as a novel antioxidant protein and offer new and important insights into its role in the OS-response and its potential use in future antioxidant therapies.

## Appendix A Supplementary material

Over-expression of Oxr1-C-Mito and Oxr1-C-Cyto constructs (A) Representative image of transfected HeLa cells with Oxr1-S-Cyto or Oxr1-S-Mito constructs. HeLa cells were immunostained with anti-myc (for Oxr1) and anti-COX IV. Oxr1-S-Mito was co-localised with mitochondrial marker COX IV with a typical mitochondrial filamentous morphology, scattered in the cytoplasm, while Oxr1-S-Cyto had a cytoplasmic diffuse pattern. Nuclei mounted with DAPI. Scale bar=20µm. (B) HeLa cells over-expressing Oxr1-C-Cyto or Oxr1-C-Mito were sub-fractionated into cytosolic and mitochondrial fractions and labeled with anti-myc (for Oxr1), cytoplasmic marker GAPDH and mitochondrial marker COX IV. The additional band seen in after Oxr1-C-Mito transfection is likely due to the cleavage of the signalling peptide in the mitochondria, as expected.

Oxr1 protein levels in N2a cells after oxidative stress treatment. (A) N2a cells were treated with 0.25 mM H_2_O_2_ for up to 2 h prior to fractionation to cytosolic and mitochondrial fractions. Each sub-cellular sample containing equivalent amount of protein was subjected to Western blot analysis with anti-Oxr1, anti-GAPDH and anti-COX IV. (B) Intensity of the 24 kD mitochondrial isoform was normalised to COX IV. Data from 3 independent experiments are shown ± SEM.

Marker of primary GCs Representative images confirming the expression of GABA(A) receptor alpha 6 (GABRA6) in granule neurons by immunocytochemistry from primary cerebellar GC culture of wild-type (WT) and bella (bel) mice. Scale bar = 10µm.

Ultrastructure of mitochondria in wild-type and bella cerebellum. TEM of cerebellar mitochondria from near end-stage *bella (bel)* and age-matched wild-type (WT) mice. Mitochondria exhibit normal and comparable ultrastructure from both genotypes. Scale-bars are as indicated.

Protein levels of mitochondrial complexes Equal amount (30 μg) of mitochondria purified from cerebella of 3 end-stage bella (bel) and age-matched wild-type (WT) mice were immunoblotted with an antibody cocktail against subunits of five ETC complexes: CI-NDUFB8, CII-SDHB, CIII-UQCRC2, CIV-MTCO1 and CV-ATP5A. No differences in expression were observed.

Measurement of ETC complex activities from wild-type and bella mitochondria Measurement of complex activities was conducted using spectrophotometry as described in the Material and Methods. All activity results were obtained from mitochondrial extractions from end-stage (P22) *bella (bel)* and age-matched wild-type (WT) mice (A) CI activity (B) CII activity (C) CIII activity (D) CIV activity (E) Citric synthase activity. Data are shown as the mean ± SEM from 3-6 replicates of each genotype and analysed by Student’s t- test.

## Footnotes

Appendix A Supplementary data associated with this article can be found in the online version at 10.1016/j.freeradbiomed.2016.03.029.
