# Supplementary figures and images for: The antioxidant protein Oxr1 influences aspects of mitochondrial morphology
Source: Free Radic Biol Med. 2016 Jun;95:255–67. doi: 10.1016/j.freeradbiomed.2016.03.029 (PMC4891067; doi:10.1016/j.freeradbiomed.2016.03.029)

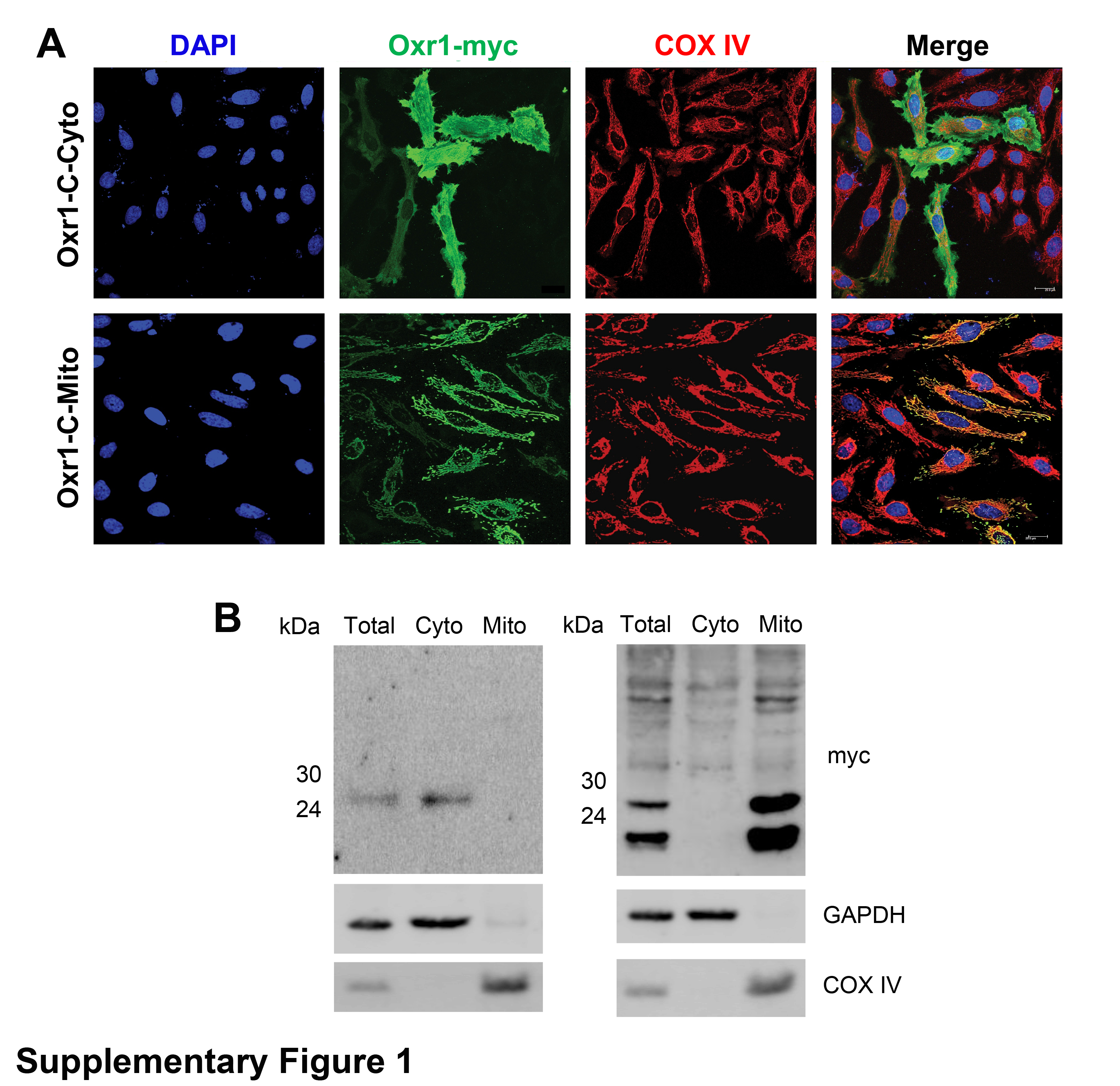

Supplement: Supplementary file 1 — Over-expression of Oxr1-C-Mito and Oxr1-C-Cyto constructs (A) Representative image of transfected HeLa cells with Oxr1-S-Cyto or Oxr1-S-Mito constructs. HeLa cells were immunostained with anti-myc (for Oxr1) and anti-COX IV. Oxr1-S-Mito was co-localised with mitochondrial marker COX IV with a typical mitochondrial filamentous morphology, scattered in the cytoplasm, while Oxr1-S-Cyto had a cytoplasmic diffuse pattern. Nuclei mounted with DAPI. Scale bar=20µm. (B) HeLa cells over-expressing Oxr1-C-Cyto or Oxr1-C-Mito were sub-fractionated into cytosolic and mitochondrial fractions and labeled with anti-myc (for Oxr1), cytoplasmic marker GAPDH and mitochondrial marker COX IV. The additional band seen in after Oxr1-C-Mito transfection is likely due to the cleavage of the signalling peptide in the mitochondria, as expected. [file mmc1.zip › mmc1.tif]

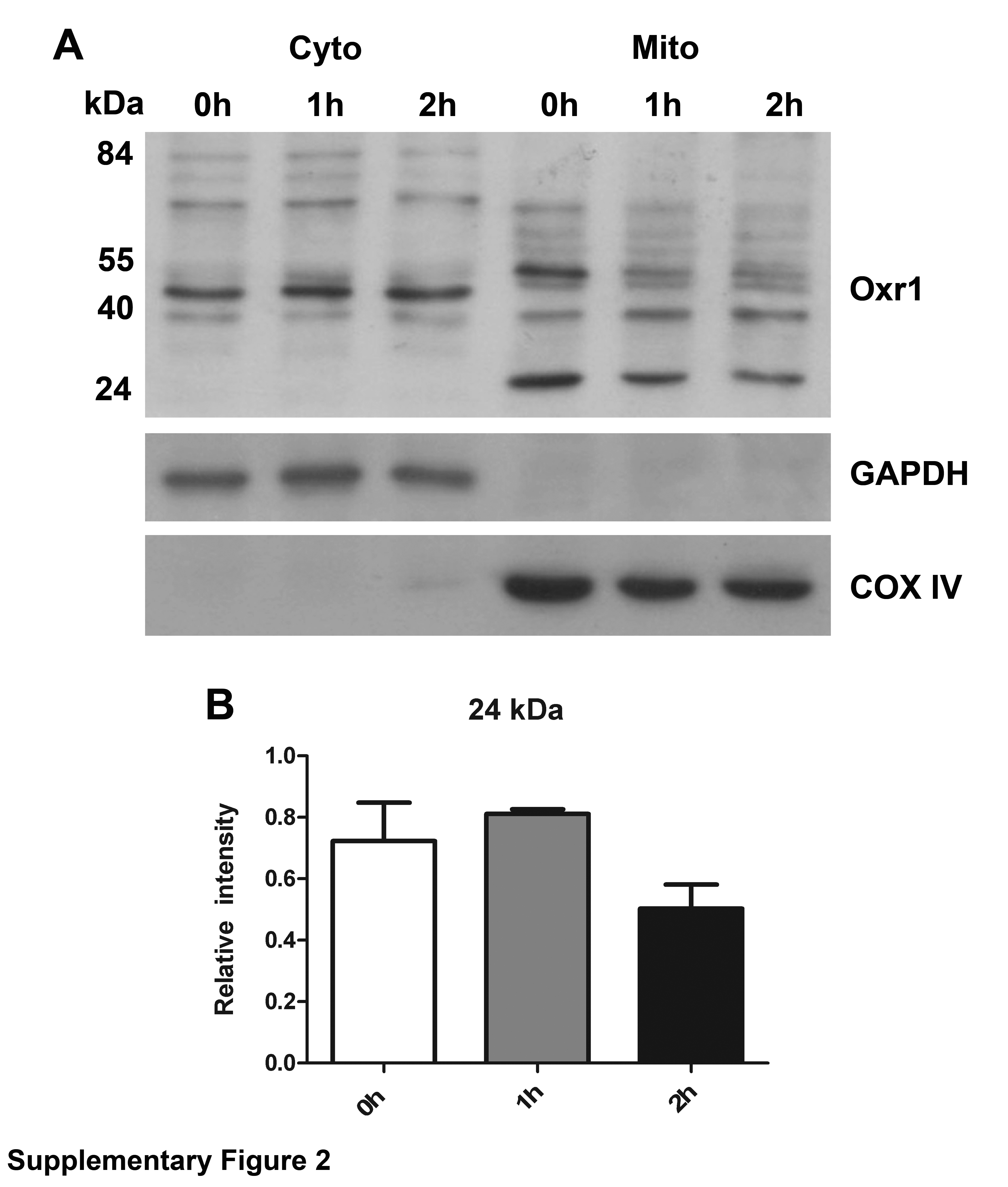

Supplement: Supplementary file 2 — Oxr1 protein levels in N2a cells after oxidative stress treatment. (A) N2a cells were treated with 0.25 mM H2O2 for up to 2 h prior to fractionation to cytosolic and mitochondrial fractions. Each sub-cellular sample containing equivalent amount of protein was subjected to Western blot analysis with anti-Oxr1, anti-GAPDH and anti-COX IV. (B) Intensity of the 24 kD mitochondrial isoform was normalised to COX IV. Data from 3 independent experiments are shown ± SEM. [file mmc2.zip › mmc2.tif]

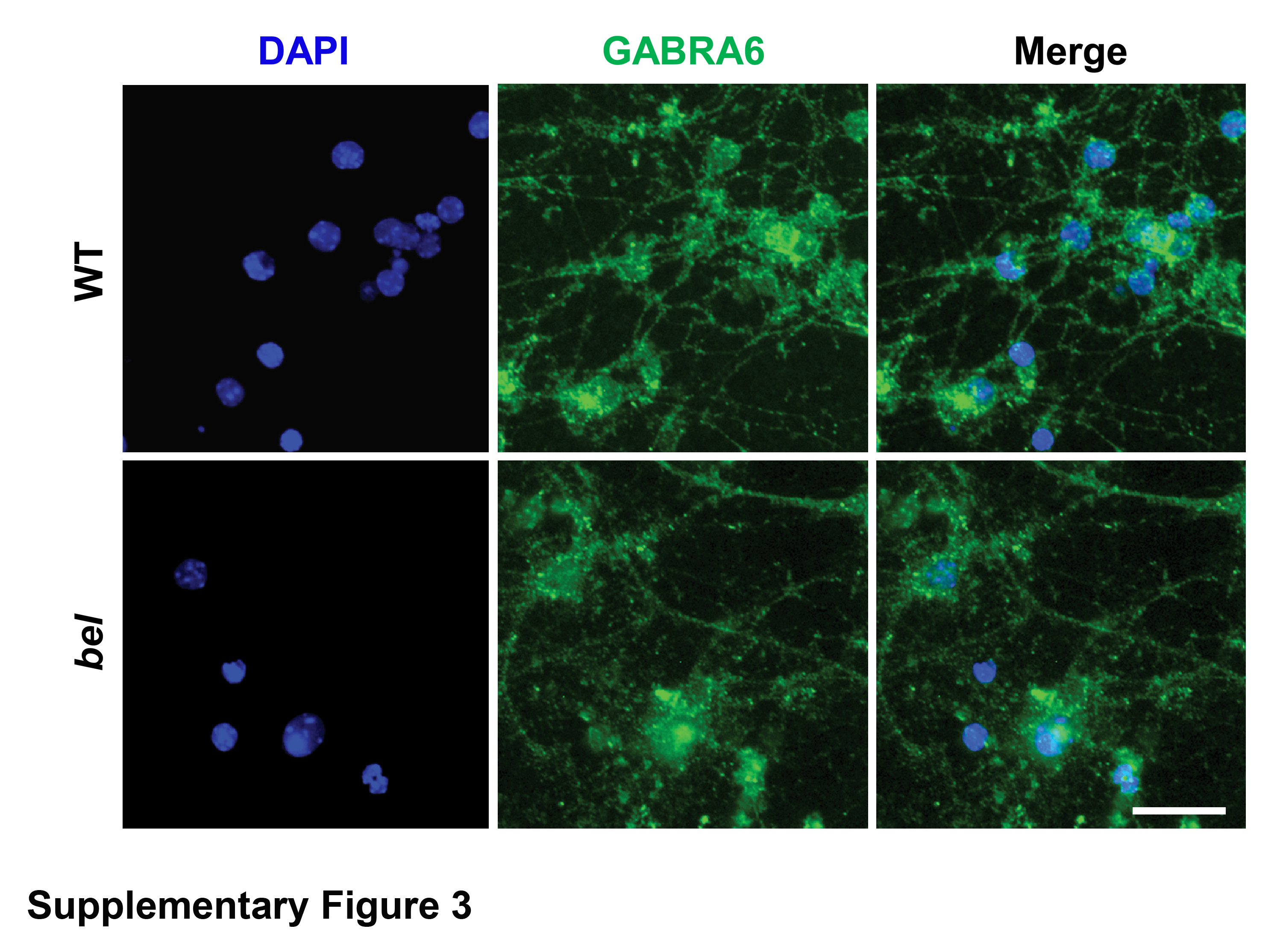

Supplement: Supplementary file 3 — Marker of primary GCs Representative images confirming the expression of GABA(A) receptor alpha 6 (GABRA6) in granule neurons by immunocytochemistry from primary cerebellar GC culture of wild-type (WT) and bella (bel) mice. Scale bar = 10µm. [file mmc3.zip › mmc3.tif]

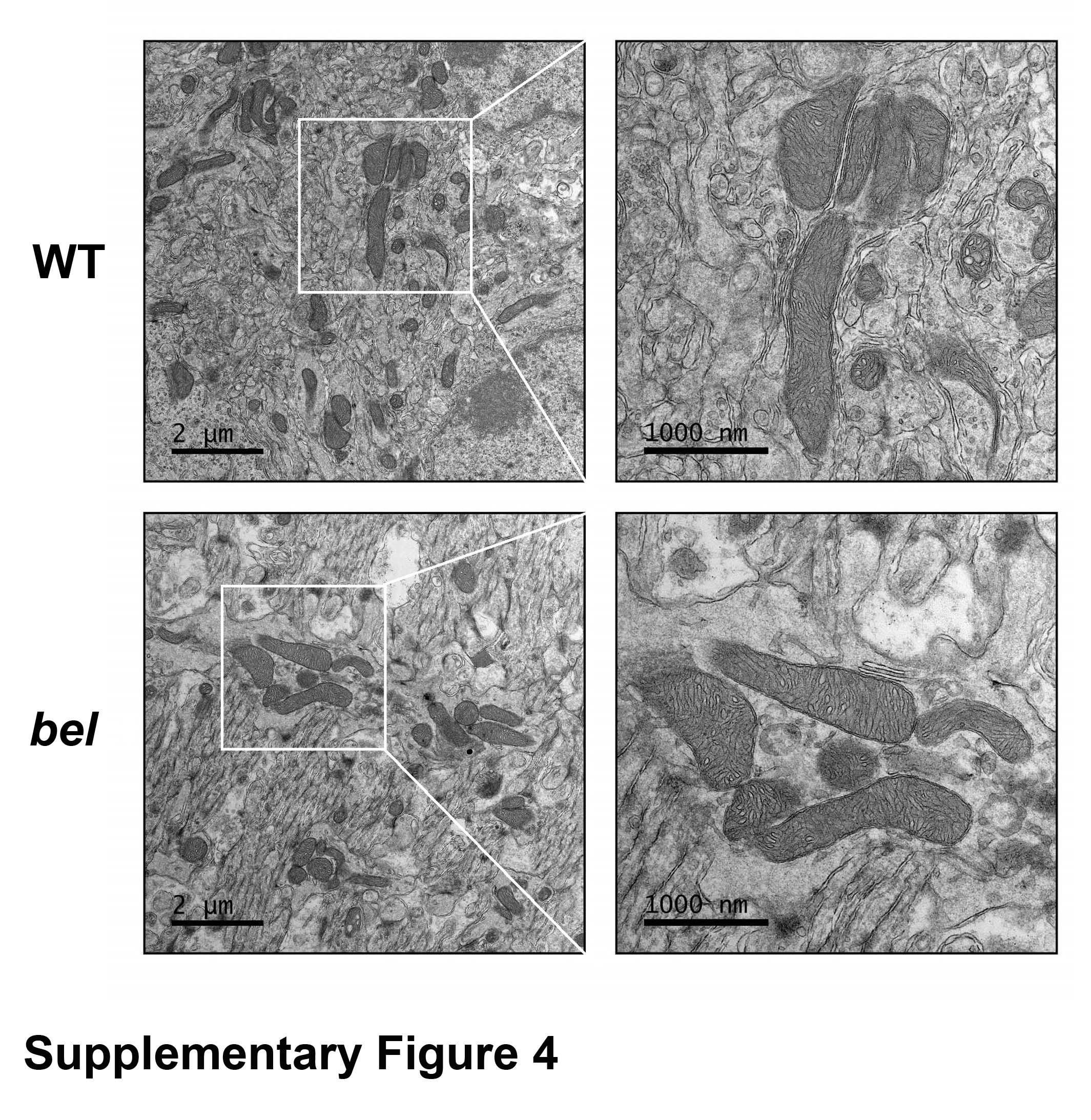

Supplement: Supplementary file 4 — Ultrastructure of mitochondria in wild-type and bella cerebellum. TEM of cerebellar mitochondria from near end-stage bella (bel) and age-matched wild-type (WT) mice. Mitochondria exhibit normal and comparable ultrastructure from both genotypes. Scale-bars are as indicated. [file mmc4.zip › mmc4.tif]

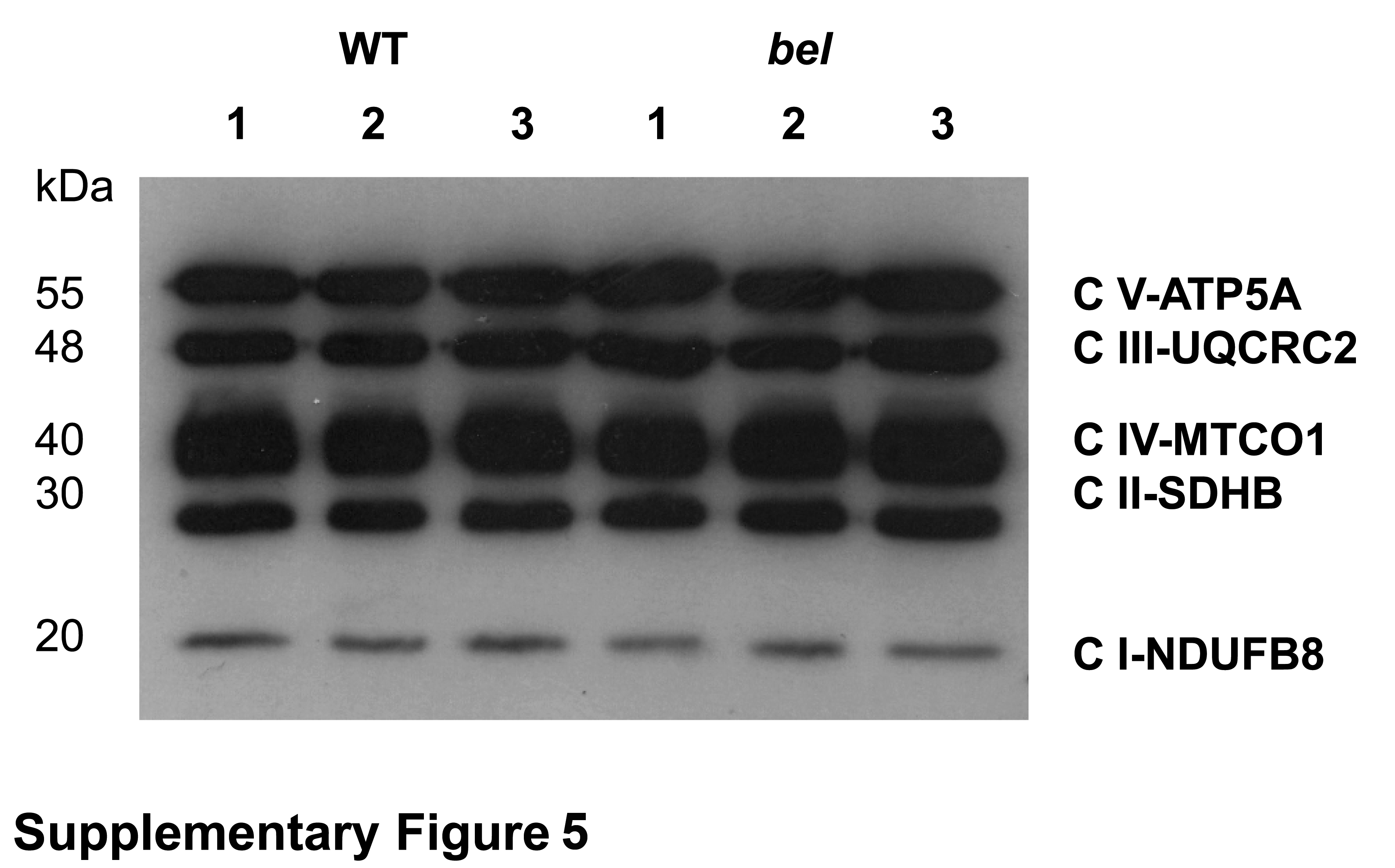

Supplement: Supplementary file 5 — Protein levels of mitochondrial complexes Equal amount (30 μg) of mitochondria purified from cerebella of 3 end-stage bella (bel) and age-matched wild-type (WT) mice were immunoblotted with an antibody cocktail against subunits of five ETC complexes: CI-NDUFB8, CII-SDHB, CIII-UQCRC2, CIV-MTCO1 and CV-ATP5A. No differences in expression were observed. [file mmc5.zip › mmc5.tif]

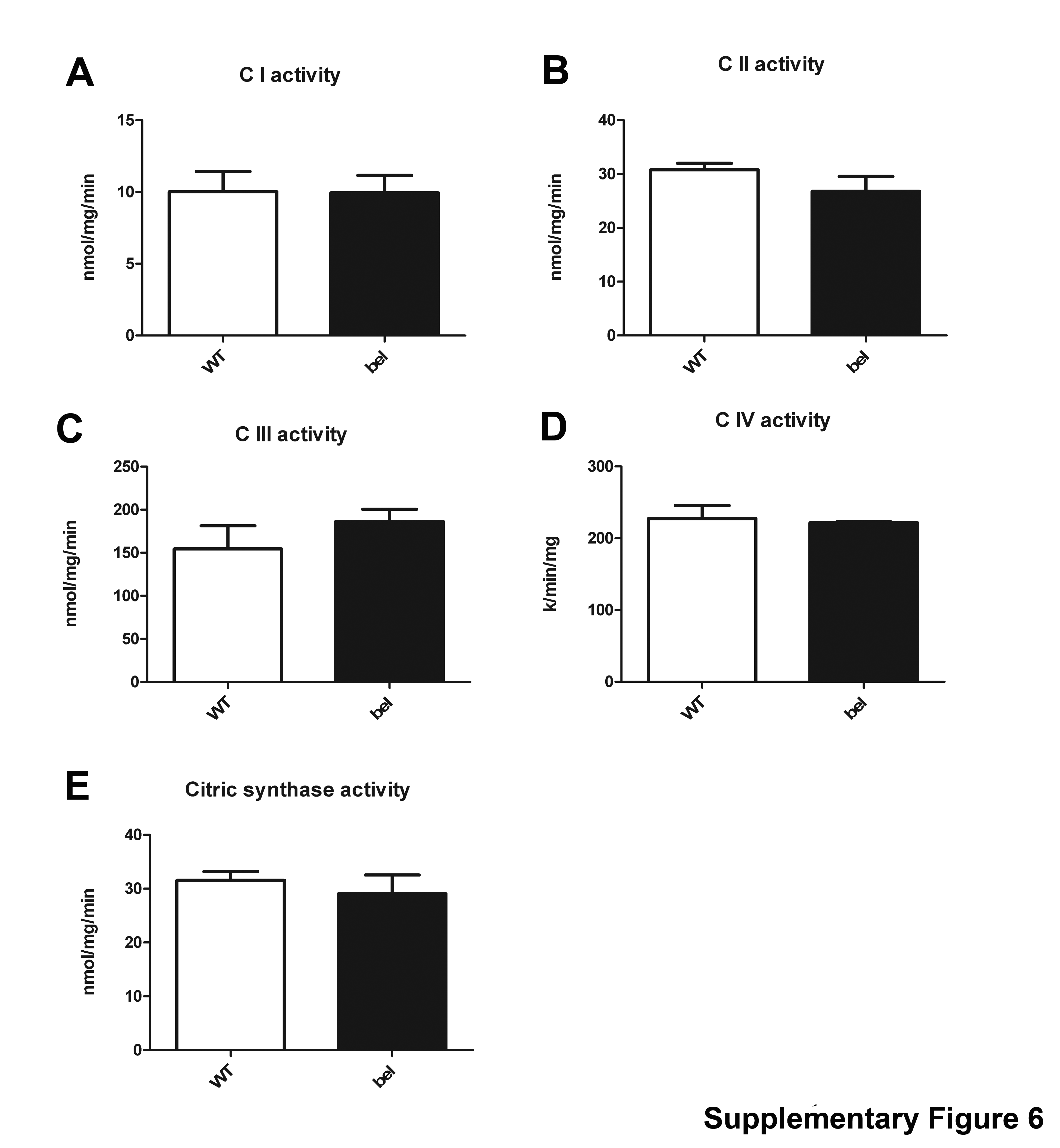

Supplement: Supplementary file 6 — Measurement of ETC complex activities from wild-type and bella mitochondria Measurement of complex activities was conducted using spectrophotometry as described in the Material and Methods. All activity results were obtained from mitochondrial extractions from end-stage (P22) bella (bel) and age-matched wild-type (WT) mice (A) CI activity (B) CII activity (C) CIII activity (D) CIV activity (E) Citric synthase activity. Data are shown as the mean ± SEM from 3-6 replicates of each genotype and analysed by Student’s t- test. [file mmc6.zip › mmc6.tif]
